# Supplementary material for: Length of hospital stay and associated factors among heart failure patients admitted to the University Hospital in Northwest Ethiopia
Source: PLoS One. 2022 Jul 22;17(7):e0270809. doi: 10.1371/journal.pone.0270809 (PMC9307162; doi:10.1371/journal.pone.0270809)
Supplement: S1 Table — (PDF) [file pone.0270809.s001.pdf]

S1 Table. Correlations between patient characteristics and LOS among AHF patients.

Variable	Pearson rho	Spearman's rho	P-value
Gender		-0.085	0.171
Age	-0.130		0.036*
Types of HF		-0.060	0.330
Causes of HF		0.151	0.014*
Precipitating factors of HF		0.181	0.003*
Asthma		0.065	0.296
Sepsis		-0.029	0.641
Cardiogenic shock		0.030	0.625
Diabetes mellitus		0.082	0.182
Anemia		-0.116	0.60
Dyslipidemia		0.109	0.078
Hypertension		-0.047	0.446
Atrial fibrillation		0.011	0.857
Renal disease		-0.040	0.523
Stroke		-0.052	0.405
Thyroid disease		0.077	0.213
Degenerative valvular heart disease		-0.092	0.136
Cancer comorbidity		-0.056	0.364
HIV comorbidity		-0.145	0.019*
Tuberculosis		-0.088	0.155
Cor pulmonale		-0.048	0.436
Ischemic heart disease		0.070	0.256
Rheumatic heart disease		-0.129	0.037*
Chronic obstructive pulmonary disease		-0.078	0.206
community acquired pneumonia		0.180	0.003*
Dilated cardiomyopathy		0.131	0.034*
Number of comorbidities	0.286		$< 0.001^{**}$
New York Heart Association functional class		0.138	0.026*

Systolic blood pressure		-0.114	0.064
Diastolic blood pressure		-0.118	0.055
Respiratory rate		-0.198	0.001*
Heart rate		0.028	0.656
Temperature	0.073		0.238
Left ventricular ejection fraction		-0.036	0.556
Respiratory rate		0.198	0.001*
Serum creatine		-0.080	0.198
Hemoglobin		-0.011	0.860
Serum sodium	0.032		0.602
Serum potassium		0.238	< 0.001**
Dyspnea at rest		0.053	0.393
Dyspnea on exertion		0.006	0.925
Easy fatiguability		0.004	0.947
Atrial fibrillation		-0.006	0.918
Peripheral edema		0.067	0.282
Elevate jugular venous pressure		0.143	0.020*
Cardiomegaly		0.001	0.989
Third heart sound (S_3 gallop)		0.168	0.006*
Orthopnea		0.055	0.371
Paroxysmal nocturnal dyspnea		0.145	0.019*
Neck vein distension		0.053	0.391
Murmur		0.131	0.034*
Furosemide		-0.003	0.960
Spironolactone		0.080	0.195
Digoxin		0.40	0.520
Enalapril		0.106	0.087
Metoprolol		-0.036	0.557
Atenolol		-0.124	0.045*
Aspirin		0.094	0.128
Warfarin		-0.055	0.376

Atorvastatin		0.153	0.013*
Unfractionated heparin		-0.079	0.201
Amlodipine		-0.076	0.222
Dopamine		-0.115	0.063
Clopidogrel		0.095	0.125
Number of medications		-0.175	0.004*

HF, heart failure; HIV, Human Immunodeficiency Virus

*Correlation is significant at $p \leq 0.05$ (2-tailed).

**Correlation is significant at $p < 0.001$ (2-tailed).
