# Supplementary material for: Length of hospital stay and associated factors among heart failure patients admitted to the University Hospital in Northwest Ethiopia
Source: PLoS One. 2022 Jul 22;17(7):e0270809. doi: 10.1371/journal.pone.0270809 (PMC9307162; doi:10.1371/journal.pone.0270809)
Supplement: S1 Annex — (PDF) [file pone.0270809.s002.pdf]

S1 Annex. Data abstraction format of AHF patients.

Code: ___________________________

Section I: Socio-demographic Characteristics

Gender Male Female **Age: _______________**

Section II: Clinical Characteristics at Admission

Types of HF New onset ADCHF

NYHA functional Class I II III IV

Vital signs at admission

Systolic blood pressure __________ (mmHg) Diastolic blood pressure ____ (mmHg)

Pulse rate ___________ (bpm) Respiratory rate ___________ (bpm)

Body temperature __________ (°C)

Sign and symptoms at admission

Cardiomegaly Neck vein distension Orthopnea

S3 gallop (third heart sound) PND

Easy fatiguability Peripheral edema Dyspnea at rest

Dyspnea on exertion Murmur Elevated JVP

Electrocardiogram finding

AF

Echocardiogram finding

LVEF: ________________ (%)

Laboratory values

Serum Na+ ________ (mEq/L) Serum K+ _________ (mEq/L)

Hemoglobin _________(g/dL) Serum creatinine __________(mg/dL)

Precipitating factors of AHF at Admission

IHD Anemia AF

CAP HTN DCMP

TB Thyroid disorder Renal disease

Non adherence to medical regimen Undefined

Other: ______________________

Underlying causes of AHF at Admission

CRHD Cor pulmonale DVHD DCMP

HTN IHD Stroke Anemia

AF COPD Other: _________________________

Co-morbidity

IHD AF Asthma DCMP

Cancer Renal disease COPD Diabetes mellitus

Dyslipidemia HIV/AIDS Thyroid disorder

HTN TB CRHD Stroke

DVHD CAP Dyslipidemia Sepsis

Cardiogenic shock Cor pulmonale Other: __________

Number of comorbidities

Section III: Medication Assessment

Medications given for the management of AHF during the hospital course

Furosemide Spironolactone Digoxin Metoprolol

Atenolol Enalapril Aspirin Warfarin

Atorvastatin Unfractionated Heparin Amlodipine Propranolol

Dopamine Clopidogrel Other: _________________________

Number of medications: ________________________

Use of Mechanical ventilation (Intranasal oxygen therapy) Yes No

Medications given during discharge

Furosemide	Spironolactone	Digoxin	Metoprolol
Atenolol	Enalapril	Aspirin	Warfarin
Atorvastatin	Amlodipine Propranolol	Clopidogrel	

Section IV: Outcome

Date of admission: ________________ Date of Discharge: ______________

Length of hospital stay: _______________________
