# Supplementary material for: Length of hospital stay and associated factors among heart failure patients admitted to the University Hospital in Northwest Ethiopia
Source: PLoS One. 2022 Jul 22;17(7):e0270809. doi: 10.1371/journal.pone.0270809 (PMC9307162; doi:10.1371/journal.pone.0270809)
Supplement: Attachment — Submitted filename: Response to Reviewers.docx [file pone.0270809.s003.docx]

**Rebuttal letter**

Dear editor,

Thank you for your sincere comments. Please find our response to your concerns in the previously submitted manuscript in PLOS ONE Journal.

1. Please revise and shorten the Title to make it concise and informative.

Dear editor, the title was revised and shortened from “*Length of hospital stay and associated factors among heart failure patients admitted to the medical ward of University of Gondar Comprehensive specialized Hospital, Northwest Ethiopia: A Cross sectional study*” to “***Length of hospital stay and associated factors among heart failure patients admitted to the University Hospital in Northwest Ethiopia***”

2. Please do English editing first--too many English errors.

Dear editor, We have made English editing to the best of our capacity and please consider our revised manuscript to proceed in your esteemed journal.
