# Supplementary material for: Length of hospital stay and associated factors among heart failure patients admitted to the University Hospital in Northwest Ethiopia
Source: PLoS One. 2022 Jul 22;17(7):e0270809. doi: 10.1371/journal.pone.0270809 (PMC9307162; doi:10.1371/journal.pone.0270809)
Supplement: Attachment — Submitted filename: Response to reviewers2.docx [file pone.0270809.s004.docx]

**Response to reviewers**

Reviewer #1:

The Authors have adequately replied to previous editor comments. The paper is interesting, however it's not easy to read this manuscript, the intro is too long and also data presentation is complex with a lot of data and numbers. It's unclear why the use of diuretics, the type of underlying disease (i.e CAD, VHD or DCM), the time from symptom onset to hospital admission and finally the age, didn't enter in the multivariate analysis

**Dear Reviewer:** Thank you for your constructive comments. We have tried to address your concerns and we found most of them as helpful to the paper improvement.

- With regard to length of the introduction section, we have reduced some sentences which are not much related with length of hospital stay of heart failure patients.
- With data presentation and numbers, we understand it is appropriate concern having more numerical presentation may not be attractive to the reader. But we have kept them as it is since we believe reducing the data and numerical values may not give the full picture of the extent of association of variables with patients’ length of hospital stay.
- Concerning the variables in the multivariate analysis, we included the missed variables as per your comments. Use of diuretics is not satisfied the requirement of the multivariate analysis since it has no significant association with length of hospital stay in the univariate analysis and was not considered for multivariate analysis. Age is already included in the multivariate analysis though it was not found to have significant association. The underlying diseases included to the multivariate analysis and we did not found association with length of hospital stay by themselves. But it affected slightly the extent of association of other variables with little numerical variation without change of direction of association. Thus, the table is amended considering the new variable added to the multivariable analysis.
