# Supplementary material for: Length of hospital stay and associated factors among heart failure patients admitted to the University Hospital in Northwest Ethiopia
Source: PLoS One. 2022 Jul 22;17(7):e0270809. doi: 10.1371/journal.pone.0270809 (PMC9307162; doi:10.1371/journal.pone.0270809)
Supplement: Attachment — Submitted filename: Response to Reviewerss.docx [file pone.0270809.s005.docx]

**Response to reviewers**

Reviewer #3:

We have made a review for Manuscript Number PONE-D-21-01805R2; entitled: Length of hospital stay and associated factors among heart failure patients admitted to the University Hospital in Northwest Ethiopia. The current manuscript has critical points:
1- The very limited sample size of just 263 patients and the high number of variables incorporated in the analysis compared to the small sample size. This would greatly affect the confidence with the results.
2- The authors did not incorporate important variables of heart failure assessment in modern cardiology like Echocardiography parameters, natriuretic peptides level, etc...
3- Variables described by the authors like third heart sound, etc are considered diagnostic variables of decompensated heart failure.
4- Based on the prior comments, the clinical implications of the current manuscript will not be clear and practical.
5- Extensive editing is required.
**Dear Reviewer:** Thank you for your constructive comments and concerns. We appreciate for your concerns.

Regarding the very limited sample size and the high number of variables incorporated in the analysis compared to the small sample size this might affect the confidence with the results. But we have tried to include all CHF patients during the study period. The prospective nature of the study design would increase the confidence of the results. Besides, we have disclosed the small size of the study may affect the generalization of the results. Thus, we have informed future researchers to consider the limitation of our study. Please note our concern was including the variables which are relevant to the length of hospital stay. However because of time and financial constraints we can’t include a large number of samples in our study. That is why these variables become large comparing to the small sample size.

Regarding the incorporation of important variables of heart failure assessment in modern cardiology like Echocardiography parameters, natriuretic peptides level. The reason that these variables were not included in the study was such Echocardiographic parameters such as natriuretic peptides level were not done in the study setting.

Concerning the variables like third heart sound, etc are considered diagnostic variables of decompensated heart failure. In the study, not only new onset acute heart failure patients but also those who were admitted with acutely decompensated chronic heart failure patients were included, The impact of the clinical features of these patients on length of hospital stay were assessed because these variables were included in the analysis.
Regarding the clinical implications of the manuscript, we believe that the finding of study will have a role in clinical scenarios by providing data regarding the length of hospital stay and factors influencing length of hospital stay in patients with HF. Specifically, factors determine the current status of length of hospital stay and associated factors among hospitalized acute heart failure patients in the medical ward of the University of Gondar Comprehensive Specialized Hospital in Ethiopia were provided to the clinical practitioners and researchers. Moreover, clinicians would be aware of the clinical features contributing to the longer hospital stay and implementation of interventions or strategies that could reduce the heart failure patient’s hospital stay is necessary.
