# Supplementary material for: Length of hospital stay and associated factors among heart failure patients admitted to the University Hospital in Northwest Ethiopia
Source: PLoS One. 2022 Jul 22;17(7):e0270809. doi: 10.1371/journal.pone.0270809 (PMC9307162; doi:10.1371/journal.pone.0270809)
Supplement: Attachment — Submitted filename: Response to reviewers 4.docx [file pone.0270809.s006.docx]

**Response to reviewers**

Reviewer #4:

Dr Tekle MT et al. reported clinical factors associated with length of hospital stay in patients hospitalized for acute heart failure. Unfortunately, the manuscript was poorly written, and require adequate quality of English edition. My major concern is that length of hospital stay may be influenced by different health insurance system, hospital capacity, patterns of clinical practice and physicians. Therefore, generally, this topic may lack of generalization, and need to be explored in national database or multicenter cohorts. In this regard, the interpretation of their results may be challenging. For example, their results showed that factors precipitated to acute heart failure were associated with length of hospital stay; however, various types of precipitating factors (i.e., acute coronary syndrome, infection, non-adherence etc) exist and may influence length of hospital stay. Patients with acute heart failure precipitated by acute coronary syndrome were more likely to require a longer hospital stay than those precipitated by non-adherence since these patients need a cardiac catheterization during hospital stay. Please describe your findings more precisely.
Furthermore, not surprisingly, their results showed that sicker patients with acute heart failure supported by more frequent comorbidities and/or more severe congestion were more likely to need hospital care longer. I am wondering about novel findings of the current study. If they use database from acute heart failure patients in Ethiopia as a representative of developing countries, which factor is specific for patients in developing countries.

**Dear Reviewer:** Thank you for your constructive comments and concerns. We have tried to address your concerns and we found most of them as helpful to the paper improvement.

Thank you for your constructive comments which is helpful for our paper quality. Regarding revising of the manuscript for an English writing, we have made English editing on the revised manuscript. For example in the introduction section we have tried to reduce its length by removing less relevant statements and some redundant expressions. Additionally, in the result section in order to increase its clarity we have made some modification on it.

As you have described in addition to the individual patient’s characteristics length of hospital stay might be influenced by organizational, health insurance system, hospital capacity, patterns of clinical practice and physician related factors. However, because of inconveniences, resources and time constraints we were not able to address these factors. For our study this will be a weakness and we have tried to explain in the limitation section of the revised manuscript.

Other concerns were raised as follows:

1. Overall, in introduction section, interpretation of some texts was confusing. Length of hospital is a result of a hospital care. Authors described “decreased LOS results in reduced risk of medication side effects and infection, lower mortality rates, increased hospital profit with more efficient bed management, and better treatment outcomes.” However, generally, low risk of medication side effects and infection may lead to a short hospital stay.

**Dear reviewer**: Thank you very much! On the introduction section by deleting some confusing and repeated sentences we have tried to make some modification on the revised manuscript.

2. I would disagree with author description showing that a longer hospital stay was associated with poor clinical outcomes. In some countries, patients are up-titrated to maximum tolerated doses of guideline recommended heart failure medications during their hospital stay, leading to favorable outcomes. Please comment on this issue.

Thank you for your comment. As you mentioned in management of AHF to get good clinical outcomes patients are up-titrated to maximum tolerated doses of guideline recommended but we use this statement to mean that AHF patients who stayed longer in the hospital are more likely exposed to experiencing unfavorable clinical outcomes such as developing life-threatening medical complications, hospital acquired infections, medication side effects, increased readmission, and high mortality.

3. Acute heart failure is characterized by heterogeneous phenotypes, and, sometimes, it is challenging to diagnose of this cardiac syndrome. Please describe detailed information about acute heart failure definition in the methods section, citing a relevant paper.

Thank you. Based on your comment we have included a sentence which describe about acute heart failure.

4. Please explain heart failure medications in detailed. The term “number of medications” is confusing. Do “medications” indicate specific treatments for heart failure or general drugs such as proton pump inhibitor and antiplatelet therapy? In addition, do “number of medications” mean number of drug tablets?

Thank you for the comment. In the study, patients were treated with heart failure medications such as metoprolol, digoxin however the term number of medications was used to describe total number of medications (including other drugs) such as antiplatelet therapy like aspirin. Additionally, this term was used to mean the total number of medications taken by the individual HF patient during the hospital course.

5. Spearman is not appropriate for looking at correlation between continuous variable and categorical variable. Please remove these results or analyze it with correct statistical approach.

Thank you! While we appreciate the reviewer’s feedback, we respectfully disagree. Most statistical evidence explained that spearman rank correlation coefficient is used to measure the strength of the linear association between two variables specifically strength of linear association between one continuous dependent variable and one ordinal, or categorical independent variable. Because of this we did not reanalyze the data and modify the former results.

6. Please draw lines between rows or columns in each table. Additionally, in Table 1, some important variables are lacking. Please describe prevalence of comorbidities or precipitating factors, which may help reader understand overall patient characteristics in these patients with acute heart failure.

Thank you based on your comment we draw lines between rows/columns for each table. The most frequent comorbidities were already described in table 1 while precipitating factors of AHF were written in text.

7. Again, acute coronary syndrome is, generally, a high prevalence and important precipitating factor for acute heart failure patients. Why author do not include this factor in groups of precipitating factors?

Thank you for your concerns! In the study, IHD which is manifested with chronic stable angina and the acute coronary syndrome (unstable angina, non–ST, and ST-segment elevation myocardial infarction) was the leading co-morbid conditions. However majority of the patients were admitted with undefined precipitating factors and of the defined ones’ community acquired pneumonia and atrial fibrillation were the leading precipitating factors of HF. Because of this we did not include acute coronary syndrome groups of precipitating factors.

8. How did authors select variable associated with length of hospital stay in multivariable model? With backward or forward selection?

Thank you for the comment. Variables were selected using Enter (simultaneous regression) method.

9. Presence of elevated jugular venous pressure and/or paroxysmal nocturnal dyspnea were associated with a longer hospital stay; however, presence of III heart sound or lower admission respiratory rate were associated with a shorter hospital stay. Although all variables may express the severity of congestion, why do authors have inconsistent results?

Thank you very much for your constructive comment and concerns. As you have described all the variables which shows severity of congestion were not associated with a longer length of hospital stay. In the study for minimizing data inconsistency certain measures were tried to be undertaken. For example during data collection by using a pretested abstraction format data were collected by three trained nurses. Additionally, the collected data were edited; cleaned, coded, entered, and analyzed using SPSS 21. Even though such measures were undertaken our data we were not able to be consistent and we have displayed the results of the study as they were.

10. All patients were hospitalized for acute heart failure, however, there was quite low number of patients having guideline-directed heart failure medications; for example, only <25% of patients were prescribed enalapril at discharge. Surprisingly, some patients (17.5%) took metoprolol at discharge, but none took other beta-blocker drugs such as carvedilol and bisoprolol, which have been proven to be superior to metoprolol and are used in routine clinical practice. I am convinced that the different management of hospitalized heart failure may limit generalization of their findings.

Thank you for your constructive comment. As you have described, the study showed that only few AHF patients were treated based on guideline-directed heart failure medications. This might be due to the unavailability of these superior and highly recommended heart failure medications in the study setting.

11. How about length of hospital stay across heart failure phenotypes (i.e., reduced ejection fraction, mid-range ejection fraction and preserved ejection fraction) since different ejection fraction is not only a marker of systolic function, but also has different underlying causes, management and treatments, which might lead to different length of hospital stay.

Thank you very much for your concern. As you have described LOS might be influenced by a different heart failure phenotypes, underlying causes, management, and treatments. Despite the focus of this study was to identify factors associated with prolonged length of hospital stay, it was conducted among all hospitalized heart failure patients who were not stratified into a different heart failure type (i.e., the patients were not classified as systolic heart failure, diastolic heart failure or heart failure with reduced ejection fraction, etc.). Additionally, in the present study ejection fraction was not taken as unique marker of systolic function. Rather other markers of systolic function such as shortness of breath at rest or on exertion, orthopnea, easy fatigability, peripheral edema were under consideration.

12. Please check number of percentages in Figure 1.

Thank you. Based on your comment we have checked all percentages and they were correct.

13. Please revise the manuscript adequately for an English medical journal. Overall, this paper is not relevant; for example, length of introduction section, some redundant expressions.

We have tried to make English editing on the revised manuscript and repetitive explanations were reduced from the introduction.

Reviewer #5: The authors' purpose is valuable and this should be emphasized to begin. Indeed, there are only few data available on heart failure and HF prognosis from Africa, and most are published by large, academic, university referral hospitals. What happens in smaller medical facilities or regional university hospitals is less known. The authors performed a large study that may be suitable for a broad readership. However there are major limitations to their study.

**Dear Reviewer:** Thank you very much for your constructive comments and concerns. We have tried to address your comments in the revised manuscript.

1. As acknowledged by the previous reviewers, the manuscript is rather long, with only few,( and easy to shorten) results and conclusions. It's not easy to move from one Section to another, and to understand how relevant and useful for clinical practice the results are. Limiting the purpose to a description of the study population could be an alternate. It's interesting per se for a non-African reader to know that hospital stay for acute HF may be as long as 17 days, especially in patients aged 51 years only.

Thank you very match! Based on your comment and suggestion given by previous reviewers’ we have made English editing to the best of our capacity. For this we have made modification on the introduction and result section of the revised manuscript. We have tried to improve the manuscript flow to read well for wider readership.

2. The lack of echo and natriuretic peptides is easy to understand (BNP and NTproBNP are very expensive for low income countries). This is not a problem on my opinion.
3. The reference section may largely be improved. Data from Poland, Japan, China, Germany are not required, I believe. By contrast, the authors omitted a major study on HF from Subsaharan Africa , the Thesus-Hf study ( The sub-Saharan Africa Survey of Heart Failure (THESUS-HF) prospective cohort study).

Thank you. Based on your comment we have included the finding of the Thesus-Hf study.

4. I believe we should sustain and promote African studies especially from centers who make efforts to perform academic research. May be a way to make this submission suitable for publication would be to largely shorten it and to limit results to a description of the real world description of HF in their institution.
