# Supplementary material for: Length of hospital stay and associated factors among heart failure patients admitted to the University Hospital in Northwest Ethiopia
Source: PLoS One. 2022 Jul 22;17(7):e0270809. doi: 10.1371/journal.pone.0270809 (PMC9307162; doi:10.1371/journal.pone.0270809)
Supplement: Attachment — Submitted filename: Response to reviewers 5.docx [file pone.0270809.s007.docx]

**Response to reviewers**

Reviewer #4:

Unfortunately, my concerns were not addressed, and I cannot see what point authors modify or correct exactly in their revised version of manuscript. As authors can see in my comments, a variable, “precipitating factors”, was so confusing since clinical meaning and/or prognostic value varied greatly among precipitating factors including, acute coronary syndrome, infection, arrhythmia, and poor adherence. Even though this variable was statistically associated with length of hospital stay, I cannot interpret this result.

Sadly, although authors tried to argue my concerns, they did not modify their manuscript and explain them in the “response to reviewers”. I am convinced that readers also have similar concerns, and these raised points should be addressed adequately, particularly, in the limitations section.

Additionally, I am not sure whether the revised manuscript was edited by medical English editing. For example, their description in the results section entitled, “predictors of length of hospital stay” included many redundancies.

Furthermore, in this study, heart failure was defined per the ESC guidelines; however, in their description table, authors did not show any findings of electrocardiogram, chest x-ray, and/or echocardiogram, or values of natriuretic peptide. Please present these variables.

**Dear Reviewer:** Thank you for your constructive comments and concerns. We have tried to address your concerns and we found most of them as helpful to the paper improvement.

First of all, for the crucial comments that you have given to our paper, we would like to provide our deepest heart felt gratitude. As you have described despite the “precipitating factors of AHF” was significantly associated with LOS its interpretation was so confusing. For this we have made reanalysis to the data and the multiple linear regression showed that precipitating factors of AHF specifically infection was significantly associated with prolonged LOS. Additionally, number of comorbidities and presence of PND were significantly associated with prolonged LOS.

Thank you for your constructive comments which is helpful for our paper quality. Regarding editing of the manuscript for English write-up, we have made English editing on the revised manuscript. For example in the result section in order to increase its clarity we have removed less relevant statements and some redundant expressions. Additionally, in the introduction section we made some modification.

Thank you! Despite we did not put the findings of these investigations in the description table (table 1), we try to include in the result section of the manuscript. For example findings of chest x-ray; cardiomegaly and electrocardiogram; atrial fibrillation were included in figure 1 while finding of echocardiogram; left ventricular ejection fraction was included in the text section of socio-demographic and clinical characteristics of patients.
