# Supplementary material for: Length of hospital stay and associated factors among heart failure patients admitted to the University Hospital in Northwest Ethiopia
Source: PLoS One. 2022 Jul 22;17(7):e0270809. doi: 10.1371/journal.pone.0270809 (PMC9307162; doi:10.1371/journal.pone.0270809)
Supplement: Attachment — Submitted filename: Response to reviewers 6.docx [file pone.0270809.s008.docx]

**Response to reviewers**

**Reviewer #6:**

A significant problem with the current version of the paper is the statistical analysis. First, the use of multivariate analysis is not properly mentioned in the Methods. Second, most of the variables in linear regression (see Table 3) are qualitative, which significantly affects its efficiency. It would be more logical to use other types of regression analysis, taking into account the type of input data. In any case, it is necessary to explain the reasons for choosing the multiple analysis method in Methods by discussing its limitations in the context of the peculiarities of the data. Moreover, very many indicators are included in the analysis with a fairly limited number of observations. I am not sure if presented results are correct. The principles of selection of indicators in the multivariate model, the modeling procedure need to be clarified and, most likely, corrected.
It is unclear why the following phrases are given for a number of variables in Table 3: «causes of AHF» or « precipitating factor of AHF». Perhaps this should be reflected in the Methods when describing the data concept, or in the Discussion.

**Dear Reviewer:** Thank you for your constructive comments which is helpful for our paper quality. While we appreciate the reviewer’s feedback, we respectfully disagree. Most statistical evidence explained that from the most commonly used statistical analysis linear regression analysis is suitable for a continues dependent variable which fulfill normality distribution and linearity assumptions. In our study the dependent variable ’’length of hospital stay’’ fulfill these criteria and we chose linear regression analysis and we described this in the revised manuscript. In our study most of the variables in table 3 were categorical variables with ≥ 2 categories while few variables were continuous. For example, elevated JVP was categorized as Yes/No (patients who were admitted with the presence of elevated JVP were described as yes while those who haven’t were described as no). variables such as age, number of comorbidities, number of medications, SBP, diastolic blood pressure, respiratory rate, and serum potassium were continuous. Additionally, variables which have more than two categories (precipitating factors of AHF and causes of AHF) were analyzed by converting them in to dummy variables (Yes/No). Further, categorical variables with more than two categories specifically precipitating factors of AHF and causes of AHF were analyzed by converting them in to dummy variables (Yes/No). Because of this the phrases; causes of AHF, precipitating factor of AHF were included in table 3 and we have mentioned this in the revised manuscript. Additionally, this procedure makes many indicators to be included in the analysis. For analysis variables were selected by using Enter method.
